# Duplex ultrasound as an initial imaging clue to spontaneous isolated celiac artery dissection: A case report

**DOI:** 10.1016/j.radcr.2026.09.046

**Published:** 2026-10-06

**Authors:** Chenchen Wu, Bingru Zheng, Jiashen Wu, Zhikai Xu, Yingzhe Wu, Penghui Wang, Qianqian Wu, Runming Zhong

**Affiliations:** a Department of Radiology, The Third Affiliated Hospital of Wenzhou Medical University, Wenzhou, Zhejiang Province, China; b Department of Intervention, The Third Affiliated Hospital of Wenzhou Medical University, Wenzhou, Zhejiang Province, China; c Department of Ultrasound, The Third Affiliated Hospital of Wenzhou Medical University, Wenzhou, Zhejiang Province, China

**Keywords:** Celiac artery, Dissection, Duplex ultrasound, Computed tomography angiography, Endovascular treatment, Case report

## Abstract

Spontaneous isolated celiac artery dissection is uncommon and is usually confirmed with computed tomography angiography (CTA). Duplex ultrasound may provide an initial clue when the lesion and its flow abnormalities are directly visualized. A 56-year-old woman with untreated hypertension presented with acute persistent epigastric pain radiating to the back. Color Doppler ultrasound at the celiac artery origin demonstrated an intimal flap separating the true and false lumens, with an adjacent hypoechoic component without detectable flow. Spectral Doppler sampling within the dissected segment showed a peak systolic velocity of 225 cm/s with spectral broadening. Initial CTA confirmed proximal celiac artery dissection. On short-interval repeat imaging, the maximum outer diameter at the same anatomic level increased from 8.8 mm to 9.7 mm, the true lumen became more compressed, and new false-lumen thrombosis appeared. Symptoms had not progressed; the decision to intervene was driven by imaging progression. False-lumen coil embolization and stent placement were performed. Follow-up CTA demonstrated a patent stent, preserved major branch perfusion, and no residual false-lumen opacification. In this patient, duplex ultrasound provided an initial imaging clue, whereas CTA confirmed the diagnosis, documented interval remodeling, and assessed the post-treatment anatomy.

## Introduction

Spontaneous isolated celiac artery dissection (SICAD) is an uncommon visceral arterial disorder increasingly recognized with broader use of computed tomography angiography (CTA) [1,2]. CTA remains the principal modality for confirming the diagnosis, defining true- and false-lumen morphology, evaluating branch perfusion, and detecting interval remodeling [[1], [2], [3], [4]].

Many patients can initially be managed conservatively, but there is no universally accepted management algorithm. Treatment is individualized according to clinical status, organ perfusion, and interval imaging findings [[1], [2], [3],^5^,^6^]. We describe a patient in whom duplex ultrasound provided an initial imaging clue, short-interval CTA showed progressive morphologic change despite stable symptoms, and endovascular reconstruction was followed by post-treatment CTA assessment.

## Case report

A 56-year-old woman with a history of untreated hypertension presented with sudden-onset persistent epigastric pain radiating to the back, accompanied by nausea and vomiting. She was hemodynamically stable, and the abdomen was soft without tenderness, guarding, or peritoneal signs.

The severity of pain appeared disproportionate to the abdominal examination. Pancreatic enzymes and cardiac biomarkers were within the reference ranges.

Duplex ultrasound was performed as the initial vascular assessment using a Philips EPIQ 5 ultrasound system (Philips Healthcare, Bothell, WA, USA) with a C5-1 convex transducer. Color Doppler imaging at the celiac artery (CA) origin demonstrated an echogenic intimal flap separating the true lumen (TL) from the false lumen (FL), with adjacent hypoechoic components without detectable flow in the same segment (Figs. 1A and B). Pulsed-wave Doppler was obtained with a 2.0 mm sample volume placed within the dissected segment and a 22-degree angle correction. The peak systolic velocity was 225 cm/s with spectral broadening (Fig. 1C).

**Fig. 1:**
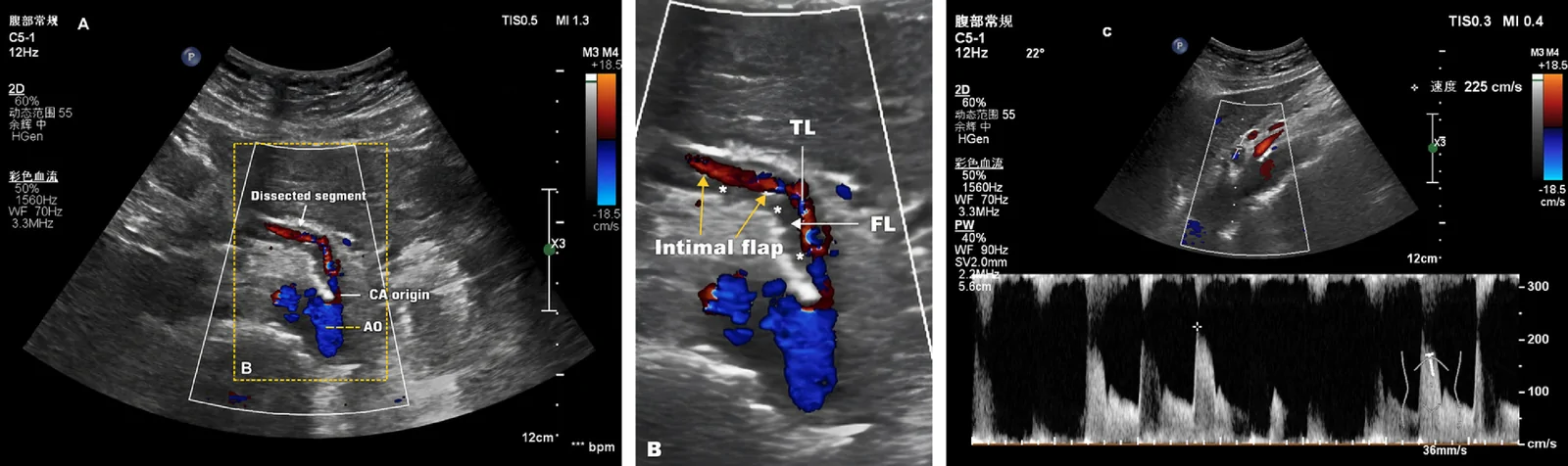
Duplex ultrasound findings at the celiac artery origin at presentation. (A) Panoramic color Doppler view shows the abdominal aorta (AO), celiac artery (CA) origin, and dissected segment. The yellow dashed box identifies the region enlarged in panel B. (B) Magnified color Doppler view from the same source image demonstrates the intimal flap (yellow arrows) separating the true lumen (TL) from the false lumen (FL). Asterisks indicate adjacent hypoechoic components without detectable flow. (C) Pulsed-wave Doppler sampling within the dissected segment, with 22-degree angle correction, shows a peak systolic velocity of 225 cm/s and spectral broadening.

The initial CTA, obtained after the duplex ultrasound examination, confirmed SICAD of the proximal CA, with an intimal flap separating the TL and FL (Fig. 2A). The maximum outer diameter at the selected anatomic level was 8.8 mm on the workstation measurement image (Fig. 2B). The common hepatic artery (CHA), splenic artery (SA), and left gastric artery (LGA) were supplied from the TL. No aortic dissection or upper abdominal organ ischemic change was identified (Fig. 2C). The laboratory and CTA findings therefore did not support pancreatitis, acute coronary syndrome, aortic dissection, or mesenteric ischemia as alternative explanations for the presentation.

**Fig. 2:**
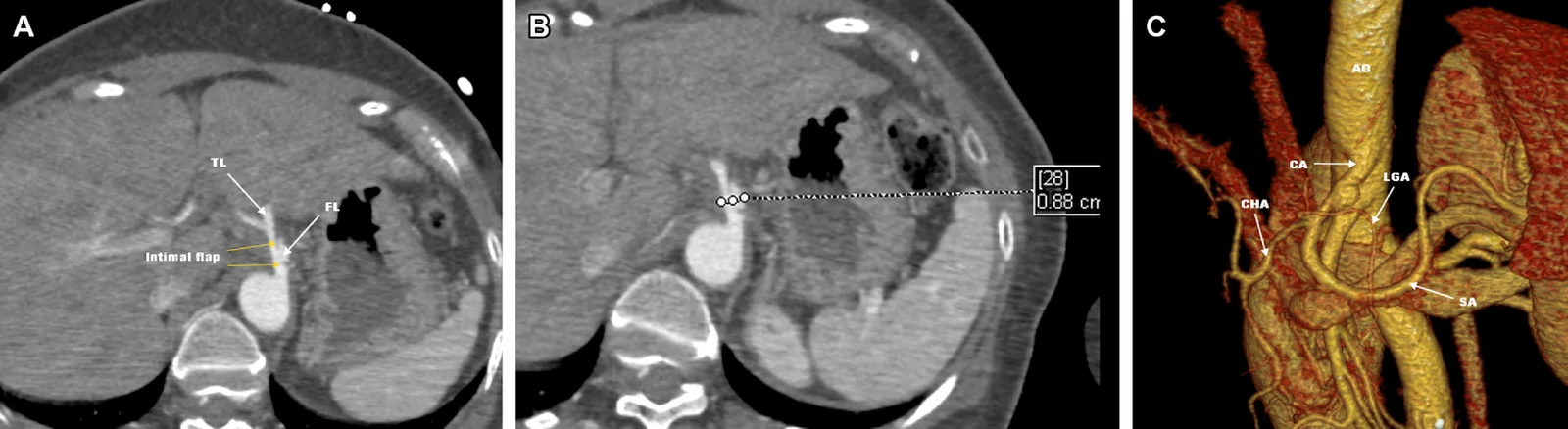
Initial computed tomography angiography of spontaneous isolated celiac artery dissection. (A) Axial contrast-enhanced CTA image demonstrates the intimal flap (yellow arrows) separating the labeled TL and FL. (B) Workstation reformatted image shows the CA origin and the native caliper measurement of the maximum outer diameter (8.8 mm; displayed as 0.88 cm). (C) Volume-rendered image shows the abdominal aorta (AO), CA, common hepatic artery (CHA), left gastric artery (LGA), and splenic artery (SA).

Initial conservative management included individualized enoxaparin at 4000 anti-Xa IU subcutaneously every 12 hours, blood pressure control, and clinical observation. Symptoms did not progress. Repeat CTA performed 7 days later nevertheless demonstrated more marked TL compression, enlargement of the FL, and new FL thrombosis (Fig. 3A). At the matched anatomic level on workstation reformats, the maximum outer diameter increased to 9.7 mm (Fig. 3B). Major branches remained perfused, and no organ ischemic change was detected (Fig. 3C and Table 1). The decision to proceed with endovascular treatment was therefore driven by interval imaging progression rather than symptom progression.

**Fig. 3:**
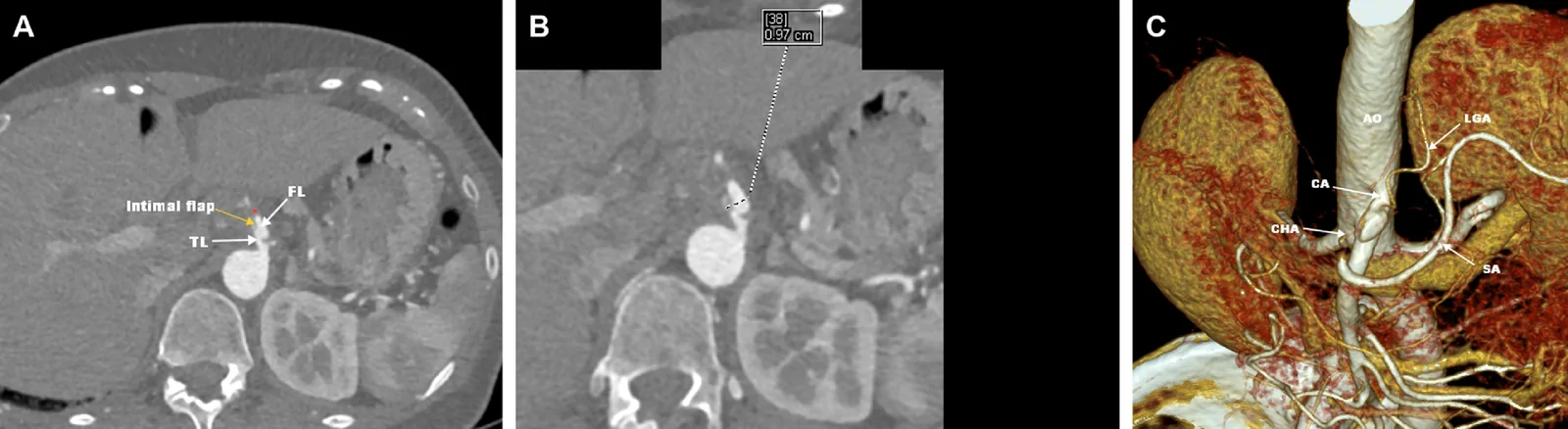
Short-interval computed tomography angiography demonstrating morphologic progression before endovascular treatment. (A) Axial contrast-enhanced CTA image demonstrates the intimal flap (yellow arrow), labeled TL and FL, and new FL thrombus (red asterisk). (B) Matched workstation reformatted image shows the CA origin and the native caliper measurement of the maximum outer diameter (9.7 mm; displayed as 0.97 cm) at the same anatomic level used in Figure 2B. (C) Volume-rendered image shows the AO, CA, CHA, LGA, and SA at the repeat examination.

**Table 1:** Serial CTA comparison before endovascular treatment.

Parameter	Initial CTA	Short-interval repeat CTA	Interval interpretation
Maximum outer diameter at the matched proximal dissected segment	8.8 mm	9.7 mm	Increased
True-lumen appearance	Compressed	More markedly compressed	Progressive compression
False-lumen appearance	Patent; no thrombus identified	Enlarged; new thrombus	Unfavorable remodeling
Major branch perfusion (CHA/LGA/SA)	Preserved from TL	Preserved from TL	Maintained
Upper abdominal organ ischemic change	Not identified	Not identified	No interval ischemic change

After the hospital's formal clinical-use approval had been obtained, endovascular treatment was performed. The guidewire was advanced through the TL, and the FL was selectively catheterized. Two 3 mm × 12 cm, 2D, 0.018 inch ZYLOX Phoenix peripheral fibered detachable coils (Zylox-Tonbridge Medical Technology Co., Ltd., Hangzhou, China) were deployed in the FL. A 6 × 60 mm self-expanding bare-metal nitinol stent (Pulsar-18 T3; BIOTRONIK AG, Bülach, Switzerland) was then deployed with its proximal end at the CA origin and its distal end in the SA, traversing the CHA and LGA ostia. The stent therefore spanned the full segment from the CA origin into the SA; its length was not a measure of the dissected segment. Completion angiography showed no residual FL opacification and confirmed patency of the CHA, LGA, and SA.

Immediately after the procedure, the patient received aspirin 100 mg once daily and rivaroxaban 10 mg once daily. Rivaroxaban was stopped at discharge, and aspirin 100 mg once daily was continued for 30 days as a post-stent regimen selected by the interventional team for this patient.

At 1-month clinical follow-up, the patient remained free of recurrent abdominal symptoms. Follow-up CTA approximately 4.5 months after the procedure demonstrated a patent stent from the CA origin into the SA, without evident stenosis, migration, or deformation. The CHA, LGA, and SA remained patent, the coils were located in the FL, and no residual FL contrast opacification was identified (Fig. 4). Retrospective review of multiplanar reconstructions and the available contrast-enhanced phases showed no reproducible distal stent filling defect. The overall clinical and imaging course is summarized in Table 2.

**Fig. 4:**
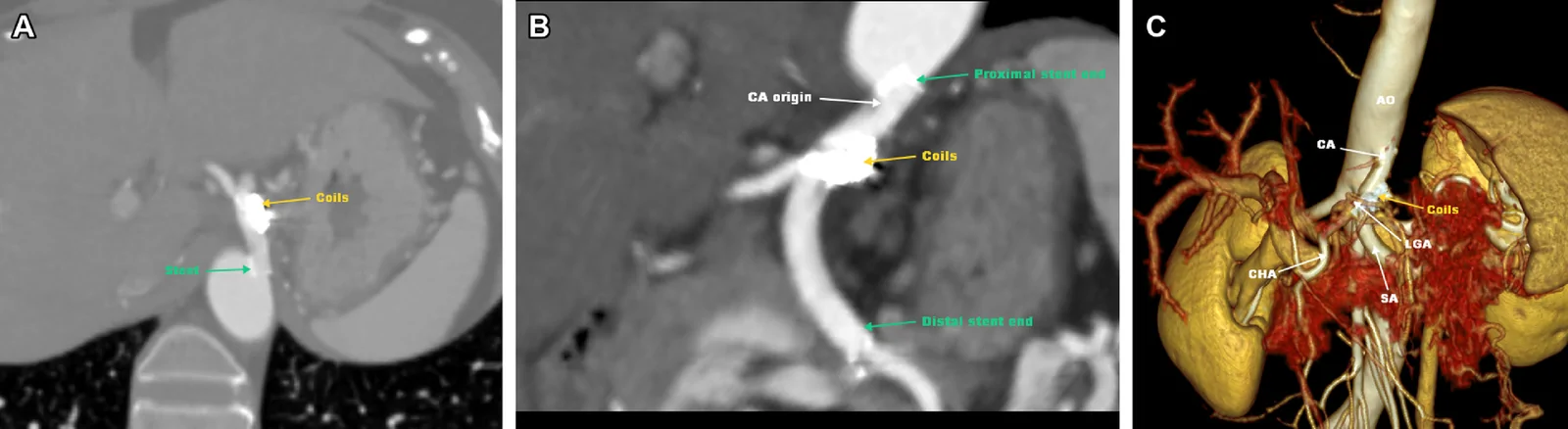
Post-treatment computed tomography angiography after false-lumen coil embolization and stent placement. (A) Axial contrast-enhanced CTA image identifies the stent in green and the embolization coils in yellow. (B) Curved planar reformatted image identifies the CA origin, proximal and distal stent ends in green, and coils in yellow; the stent extends from the CA origin into the SA. (C) Volume-rendered image shows the AO, CA, CHA, LGA, and SA after treatment; the coils are indicated in yellow.

**Table 2:** Clinical and imaging timeline.

Relative time point	Assessment or intervention	Key finding
Presentation	Clinical assessment and duplex ultrasound	Ultrasound demonstrated a flap separating TL and FL; PSV 225 cm/s with spectral broadening
Initial evaluation	CTA	Proximal SICAD confirmed; maximum outer diameter 8.8 mm; major branch perfusion preserved
Observation period	Conservative treatment	Enoxaparin, blood pressure control, and clinical observation
7-day follow-up	Repeat CTA	Maximum outer diameter 9.7 mm, greater TL compression, FL enlargement, and new FL thrombosis
Endovascular procedure	Coil embolization and stent placement	Stent extended from CA origin into SA; CHA/LGA/SA remained patent
1-month follow-up	Clinical review	No recurrent abdominal symptoms
Approximately 4.5 months	Post-treatment CTA	Patent stent, stable coils, preserved branch perfusion, no residual FL opacification, and no reproducible distal filling defect

## Discussion

This case illustrates how duplex ultrasound and CTA complemented each other. Ultrasound did not replace CTA; it prompted focused vascular evaluation by directly showing an intimal flap, separated lumens, and Doppler abnormalities within the same segment. The peak systolic velocity of 225 cm/s with spectral broadening was a descriptive, case-specific finding; validated Doppler velocity criteria for SICAD do not exist. CTA then established the diagnosis and defined the lesion and branch anatomy [1,2,4].

The decisive factor in management was the change in lesion morphology over a short interval. Most published patients with SICAD are initially treated conservatively, and treatment strategies vary among series [[1], [2], [3],[5], [6], [7]]. In this patient, symptoms did not progress and major branches remained perfused, but short-interval CTA showed increased maximum outer diameter, greater TL compression, FL enlargement, and new FL thrombosis. These directly observed interval changes, rather than symptom progression, formed the basis for escalation to endovascular treatment.

The 60 mm stent length reflected the planned landing zones rather than the length of the dissected segment itself; the device extended from the CA origin into the SA, crossing the CHA and LGA ostia. Completion angiography and follow-up CTA showed preserved branch patency. Published experience with antiplatelet and anticoagulant therapy in SICAD is heterogeneous, with no universally accepted regimen [[1], [2], [3],^5^,^6^]. Accordingly, the peri-interventional regimen and 30-day aspirin course in this case are reported as individualized choices rather than general recommendations.

Follow-up CTA showed a patent stent, preserved branch perfusion, and no residual FL opacification, and retrospective multiplanar review across the available contrast-enhanced phases identified no reproducible filling defect distal to the stent. Limitations include the single-patient design and the relatively short imaging follow-up. Longer-term observation is required to characterize durable stent patency and arterial remodeling.

## Conclusion

In this patient, duplex ultrasound provided an initial imaging clue by demonstrating an intimal flap, TL and FL, and intralesional Doppler abnormalities. CTA remained necessary for diagnostic confirmation, serial assessment of interval remodeling, treatment planning, and post-treatment evaluation.

## Ethics approval

This is a single-patient case report and does not require institutional ethics committee approval at the authors’ institution.

## Data availability

All data relevant to the report are included in the manuscript and figures. The de-identified imaging studies underlying this case report are not publicly available because of patient privacy considerations but are available from the corresponding author on reasonable request, subject to institutional policy.

## Author contributions

All authors meet the ICMJE authorship criteria. Individual contributions, described using the CRediT (Contributor Roles Taxonomy), are as follows:

**Chenchen Wu (first author):** Conceptualization; Investigation (clinical assessment and ultrasound imaging acquisition); Data curation; Visualization (figure preparation); Writing – original draft; Writing – review and editing. **Bingru Zheng:** Investigation (endovascular procedure and intra-procedural imaging); Resources (procedural materials and devices); Writing – review and editing. **Jiashen Wu:** Investigation (computed tomography angiography acquisition and interpretation); Visualization (CTA image preparation and figure assembly); Writing – review and editing. **Zhikai Xu:** Investigation (clinical management and inpatient care); Data curation (clinical and laboratory data); Writing – review and editing. **Yingzhe Wu:** Investigation (patient assessment and outpatient follow-up); Data curation (follow-up data); Writing – review and editing. **Penghui Wang:** Investigation (literature review and case workup); Methodology (referencing of reporting guidelines); Writing – review and editing. **Qianqian Wu:** Investigation (ultrasound imaging support and quality control); Visualization (ultrasound figure preparation); Writing – review and editing. **Runming Zhong (corresponding author):** Conceptualization; Methodology; Supervision; Project administration; Writing – review and editing; final approval of the submitted manuscript. All authors have read and approved the revised manuscript and agree to be accountable for all aspects of the work.

## Declaration of generative AI and AI-assisted technologies in the writing process

During the preparation of this work, the authors used OpenAI Codex and Anthropic Claude to assist with language editing, document organization, and the technical preparation of the composite figures. The composite figures were assembled from the original clinical images with scripted, non-generative tools; no generative AI was used to create or alter image content, and no AI tool interpreted the images or contributed to clinical decisions. The authors reviewed and edited all content, including figure labeling, against the source records and take full responsibility for the content of the published article.

## Patient consent

Written informed consent for publication of clinical details and accompanying de-identified images was obtained from the patient on 11 April 2026, before manuscript preparation. The original signed consent form is held by the corresponding author and is available for review by the editors on request. A separate Patient Consent Statement is submitted with this revision.
